# Application value of NIPT for uncommon fetal chromosomal abnormalities

**DOI:** 10.1186/s13039-020-00508-z

**Published:** 2020-08-28

**Authors:** Lianli Yin, Yinghua Tang, Qing Lu, Aiping Pan, Mingfang Shi

**Affiliations:** 1 Department of Clinical Laboratory, Nanning Second People’s Hospital, The Third Affiliated Hospital of Guangxi Medical University, Nanning, 530031 Guangxi China; 2 Department of Clinical Laboratory, Guangxi Hospital of Traditional Chinese Medicine, The First Affiliated Hospital of Guangxi University of Chinese Medicine, No. 89-9 Dongge Road, Nanning, 530023 Guangxi China; 3 Department of Genetic Counseling, Nanning Second People’s Hospital, The Third Affiliated Hospital of Guangxi Medical University, Nanning, 530031 Guangxi China

**Keywords:** Noninvasive prenatal testing, Birth defects, Chromosomal abnormalities

## Abstract

**Objective:**

To investigate the clinical value of noninvasive prenatal testing (NIPT) for fetal chromosomal deletion, duplication, and sex chromosome abnormalities.

**Methods:**

The study included 6239 pregnant women with singletons in the first and second trimester of pregnancy who received NIPT from December 2017 to June 2019. For pregnant women at high risk of deletion, duplication, and sex chromosome abnormalities indicated by NIPT, amniocentesis was recommended for karyotype analysis and chromosome copy number variation detection to verify the NIPT results and analyze chromosome abnormalities. Women at low risk and with no other abnormal results continued with their pregnancies.

**Results:**

Among the 6239 pregnant women who received NIPT, there were 15 cases of chromosomal deletion (12 cases confirmed by amniocentesis), 16 cases of chromosomal duplication (9 cases confirmed by amniocentesis), and 17 cases of sex chromosome abnormalities (11 cases confirmed by amniocentesis). Of these cases, 32 were finally confirmed by amniotic fluid cell karyotype analysis. The coincidence rate was 66.7% (32/48). There were no abnormalities found for the remaining low risk pregnant women during follow-up.

**Conclusion:**

NIPT has good application value in predicting fetal chromosomal deletion, duplication, and sex chromosome abnormalities. It can improve the detection rate of fetal chromosomal abnormalities, but further prenatal diagnosis is needed.

## Introduction

Fetal chromosomal abnormalities comprise one of the most important causes of birth defects. Approximately 5 million babies are born with birth defects each year worldwide, and 70% of major birth defects are caused by genetic factors. Chromosomal abnormalities and gene mutations are the main causes of genetic disorders [1]. Of all fetal chromosomal abnormalities, most are chromosomal aneuploidy abnormalities [2–4]. At present, there is no effective method of treatment for chromosomal disorders. Prenatal screening is used to identify high risk pregnant women and carry out prenatal diagnosis, early detection, and termination of pregnancy to prevent the birth of children with defects caused by chromosomal abnormalities. Traditional serological prenatal screening methods are mainly aimed at disorders such as trisomy 21, trisomy 18, and trisomy 13, although they cannot be used to screen for other fetal chromosomal abnormalities, which often results in misdiagnosis. However, the detection of fetal birth defects has gradually been popularized. More people are becoming aware that there are fetal chromosomal abnormalities other than trisomy 21, trisomy 18, and trisomy 13 [5, 6] that lead to neonatal birth defects, and the prevalence rate of these other chromosomal abnormalities is rising [7]. Compared to traditional serological screening, noninvasive prenatal testing (NIPT) has been welcomed by pregnant women and clinicians for fetal chromosomal aneuploidy screening due to its high detection rate, low false positive rate, and noninvasiveness [8, 9]. However, a large portion of the literature has focused on the study of common chromosomal aneuploidy and has rarely reported on other chromosomal abnormalities [10]. The present study aimed to explore the clinical application value of NIPT in the detection of fetal chromosomal abnormalities other than chromosomal aneuploidy.

## Materials and methods

### Subjects

A total of 6239 pregnant women who voluntarily underwent NIPT with informed consent from December 2017 to June 2019 were selected to participate in this study. The age of the pregnant women, their gestational weeks, and total number of pregnancy were recorded. An ultrasound examination was performed on each pregnant woman to confirm the number of fetuses and nuchal translucency (NT). All pregnancies were conceived naturally, and no twins or multiple twins were observed. The pregnant women were aged 18–46 years old, with an average age of 31.2 years old. The range of gestational weeks was 12–24 weeks, with an average gestational period of 18.4 weeks. Following the principles of informed consent and voluntariness, it was suggested that amniocentesis should be performed for pregnant women with NIPT results indicating a high risk of chromosomal deletion, duplication, or sex chromosome abnormalities. It was also suggested that chromosomal abnormalities should be analyzed. All pregnant women who were considered low risk and yielded no abnormal results continued with their pregnancies. All pregnant women attended follow-up. The follow-up methods included telephone follow-up and local health information system tracking. The study was carried out in accordance with the ethics committee of the Institute and Ethical Committees.

## Methods

Sample collection and processing: Peripheral blood (5 ml) was collected from each pregnant woman, and EDTA was used for anticoagulation. The plasma was centrifuged at 4 °C for 10 min at 1600 r/min and then transferred into a new 2.0 ml centrifuge tube, where it was centrifuged at 4 °C for a further 10 min at 16,000 r/min, and then stored at − 80 °C. The above steps were completed within 8 h.

Noninvasive prenatal testing: DNA was extracted using the FlexiGene DNA kit (QIAGEN, Hilden, Germany). The obtained free DNA was subjected to end-filling, the addition of A and a linker, and polymerase chain reaction (PCR) enriching. Finally, a DNA library of the corresponding sample was obtained. A pooling library was established by mixing several libraries according to a 1:1 proportion of quality. The obtained pooling library was amplified with a C-bot bridge reaction and detected with an Ion Proton Sequencing System (Life Technologies, CA, USA). The chromosomal location of each sequencing read was determined by comparing the human genome reference sequence to the GRCh37 (hg19) database. Public databases (DECIPHER, OMIM, ClinVar, ISCA) were used to interpret the data.

Karyotype analysis of the chromosomes: The NIPT indicated those pregnant women with abnormal chromosomes that underwent amniocentesis between 16 and 24 weeks of gestation with the informed consent of the women, and 15 to 20 ml of amniotic fluid were drawn for karyotype analysis. After centrifugation, 0.5 ml of each sample of the precipitate was mixed well, transferred to a bottle with amniotic fluid medium, and then placed in an incubator at 37 °C and 5% carbon dioxide for open culture. The culture medium was changed after about 7 days and cultivation was continued for 24 to 48 h; the amniotic fluid cells were observed to grow well. At this time, 60 μl of colchicine (10 μg/ml) was added into the culture bottle for 3 to 3.5 h to stop the division of the amniotic fluid cells at the middle stage. After the culture reached hypotonicity at 37 °C, fixation, preparation, banding, and staining were performed to obtain amniotic fluid cell specimens for chromosome karyotype analysis. The quality of the prepared chromosome slides was evaluated, and the metaphase was greater than 150, the number of analyzable metaphase spreads with no or very few crossovers should be > 20%, and most of the analyzable karyotype bands were > 420 and the bands were clear. Count 20 metaphases and analyze 5 of them. The karyotype was analyzed using the G-banding technique, which was performed according to the International System for Human Cytogenetic Nomenclature 2016. The G-banding were 300 bands (8p12, 8p22; 10q23, 10q25) and 400 bands (4q22, 4q26, 4q28; 5q14, 5q21, 5q23; 9p21, 9p23; 13q33), respectively.

Chromosome copy number variation (CNV) detection: Use next generation sequencing technology for CNV detection. The genomic DNA was extracted from the amniotic fluids using a Genomic DNA Extraction kit (QIAGEN, Hilden, Germany). For each sample, 10 ml of fetal amniotic fluid were collected for DNA extraction and sequencing library preparation. An Ion Proton Sequencing System (Life Technologies, Ca, USA) was used for sequencing. The results were processed using Prenatal Data Analysis Management software 3.0 (CapitalBio, Beijing, China) at 100 Kb of resolution. Public databases (DECIPHER, OMIM, ClinVar, ISCA, NCBI, UCSC) were used to explain the data.

## Results

### Baseline characteristics of subjects

The demographic characteristics of the subjects are shown in Table 1. A total of 6239 pregnant women who received NIPT were included in this study. Of the subjects, 2932 (47%) were pregnant for the first time, 2557 (41%) were pregnant for the second time, 630 (10.1%) were pregnant for the third time, and 120 (1.9%) were pregnant for more than the third time. 35 (0.6%) pregnant women had NT higher than 2.5 mm. 36.5% of the pregnant women underwent serological screening tests prior to NIPT, of which 1247 (20%) were low risk, 659 (10.6%) were high risk, and 368 (5.9%) were observed to have a serological screening index multiple of the median (MoM) value that was abnormal. The remaining 3965 (63.5%) pregnant women chose to perform NIPT directly.

**Table 1:** Baseline characteristics of the 6239 pregnant women

Age (years)	n (%)	Gestational weeks	n (%)	Number of pregnancy	n (%)	NT (mm)	n (%)	Prior screening tests	n (%)
18–25	974 (15.6%)	12–13^+6^	2051 (32.9%)	1	2932 (47.0%)	1–1.5	2807 (45.0%)	High risk	659 (10.6%)
26–35	3992 (64.0%)	14–15^+6^	3160 (50.6%)	2	2557 (41.0%)	1.6–2.0	2745 (44.0%)	Low risk	1247 (20.0%)
36–44	1247 (20.0%)	16–20^+6^	931 (14.9%)	3	630 (10.1%)	2.1–2.5	652 (10.4%)	Abnormal MoM	368 (5.9%)
> 44	26 (0.4%)	> 21	97 (1.6%)	> 3	120 (1.9%)	> 2.5	35 (0.6%)	Only NIPT	3965 (63.5%)

*NIPT* noninvasive prenatal testing, *n* number, *NT* nuchal translucency, *MoM* multiple of the median

### Chromosomal deletion

Among the 6239 pregnant women who received NIPT, a total of 15 cases of chromosomal deletions of different fragment sizes were detected, 12 of which were confirmed by amniocentesis (Table 2). The coincidence rate was 80.0%. Among the 12 confirmed cases, the results of 10 CNV detections were almost consistent with the results of the NIPT; the size of 1 deletion was slightly different from that determined by the NIPT. In addition, the CNV detection indicated an additional 2.54 Mb deletion on chromosome 4 based on the NIPT; the karyotype results were inconsistent with the NIPT results in only 1 case. The minimum fragment detected was a 0.96 Mb microdeletion; this finding was consistent with the NIPT findings. Among the 15 cases that showed a chromosomal deletion, 1 case had a slightly thicker NT (> 2.5 mm); 1 case of serological screening showed a low risk; and 9 cases were pregnant women with advanced maternal age (Age > 35 years).

**Table 2:** Karyotype analysis and CNV results of 15 cases with fetal chromosome deletion indicated by NIPT

No	Age (years)	Gestational weeks	NIPT indication	NIPT results	Karyotype results	CNV results
1	36	14^+3^	Advanced maternal age	Chr9:del(23 Mb-32 Mb), 10 Mb	Refused	Chr9:del(p21.3p21.1), 10.13 Mb
2	37	21^+3^	Advanced maternal age	Chr22:del(18.64 Mb-20.14 Mb), 1.5 Mb	Refused	Chr22:del(q11.21), 1.02 Mb
3	36	17^+1^	Advanced maternal age	Chr15:del(24 Mb-28 Mb), 5 Mb	46,XN	Chr15:del(q11.2q13.1), 4.96 Mb
4	37	16^+6^	Advanced maternal age	ChrX:del(4 Mb-19 Mb), 15 Mb	46,XN	Chr4:del(q35.2), 2.54 Mb; ChrX:del(q28), 0.26 Mb
5	40	15^+6^	Advanced maternal age	Chr5:del(127.22 Mb-130.70 Mb), 3.48 Mb	46,XN	No abnormalities
6	35	14^+5^	Only NIPT	Chr9:del(100 Mb-110 Mb), 10 Mb	46,XN,del(9)(q22q31)	Chr9:del(q22.33q31.3), 9.68 Mb
7	36	17^+1^	Advanced maternal age	Chr6:del(76 Mb-145 Mb), 69 Mb; Chr13:del(59 Mb-80 Mb), 21 Mb	46,XN	No abnormalities
8	29	14^+6^	Only NIPT	Chr22:del(18 Mb-22 Mb), 5 Mb	46,XN,inv(9)(p12q13)	No abnormalities
9	30	20^+5^	Serological screening indicated low risk	Chr22:del(18 Mb-20 Mb), 3 Mb	46,XN	Chr22:del(q11.21), 2.79 Mb
10	35	16^+5^	Only NIPT	Chr8:del(0.1 Mb-15 Mb), 15 Mb	46,XN,del(8)(p21.3p23)	Chr8:del(p23.2p21.3), 14.94 Mb
11	26	13^+6^	Only NIPT	Chr18:del(65 Mb-76 Mb), 12 Mb	46,XN	Chr18:del(q22.1q23), 12.41 Mb
12	38	22^+1^	Advanced maternal age	Chr22:del(19 Mb-20 Mb), 1.56 Mb	46,XN	Chr22:del(q11.21), 0.96 Mb
13	34	16^+5^	NT (3.2 mm)	Chr1:del(54 Mb-58 Mb), 5 Mb	46,XN	Chr1:del(p32.3p32.2), 3.42 Mb
14	39	16^+0^	Advanced maternal age	Chr22:del(18 Mb-20 Mb), 2.58 Mb	Refused	No abnormalities
15	36	17^+1^	Advanced maternal age	Chr4:del(75 Mb-81 Mb), 7 Mb	46,XN	Chr4:del(q13.3q21.21), 8.12 Mb

*NIPT* noninvasive prenatal testing, *CNV* copy number variation, *NT* nuchal translucency

### Chromosomal duplication

Similarly, chromosomal duplication was observed in 16 of the NIPT results, 9 of which were confirmed by amniocentesis (Table 3). The coincidence rate was 50.0%. The results of 3 CNV detections showed that the sizes of the fragment repeats were different from the sizes of the NIPT fragments. The CNV detection results all showed multiple segments microdeletion. One of the results of the CNV detection was a newly discovered microdeletion of about 4.65 Mb in chromosome 17. In 1 case, NIPT revealed a 78 Mb duplication of chromosome 11, while CNV detection revealed a microduplication of approximately 1 mb on chromosome 16. In another case, the karyotype results were inconsistent with the NIPT results. The results of 4 cases of CNV detection were similar to those of NIPT. Among the 16 cases that showed chromosomal duplication, 1 case of serological screening showed a high risk of trisomy 21 (≥ 1/270); 1 case of serological screening showed abnormal alpha fetoprotein (AFP) MoM; 2 cases of serological screening showed a low risk; and 5 cases were pregnant women with advanced maternal age.

**Table 3:** Karyotype analysis and CNV results of 15 cases with fetal chromosome duplication indicated by NIPT

No.	Age (years)	Gestational weeks	NIPT indication	NIPT results	Karyotype results	CNV results
1	27	18^+3^	Only NIPT	Chr14:dup(75 Mb-105 Mb), 30 Mb	46,XN,inv(14)(q24q32)	Chr14:dup(q24.3q32.33), 28.61 Mb
2	30	20^+4^	High risk of trisomy 21	Chr13:dup(27 Mb-30 Mb), 3 Mb	46,XN	Chr13:dup(q12.2), 0.46 Mb; Chr13:dup(q12.3), 0.48 Mb
3	36	15^+1^	Advanced maternal age	Chr12:dup(0.1 Mb-21 Mb), 21 Mb	46,XN,15cen+	No abnormalities
4	28	20^+2^	Serological screening indicated low risk	Chr8:dup(34 Mb-40 Mb), 7 Mb	46,XN	No abnormalities
5	33	23^+2^	Only NIPT	Chr20:dup(5 Mb-9 Mb), 4 Mb	46,XN	No abnormalities
6	37	19^+6^	Advanced maternal age	Chr8:dup(85 Mb-91 Mb), 6 Mb	46,XN	Chr8:dup(q21.2), 0.52 Mb
7	28	14^+6^	Only NIPT	Chr20:dup(47 Mb-58 Mb), 11 Mb	46,XN	No abnormalities
8	39	14^+2^	Advanced maternal age	Chr12:dup(1 Mb-33 Mb), 32 Mb	Refused	Chr12:dup(p13.33p12.1), 23.6 Mb
9	36	17^+5^	Advanced maternal age	Chr11:dup(97 Mb-125 Mb), 29 Mb	46,XN,dup(11)(q21q23)	Chr11:dup(q21q23.3), 20.12 Mb; Chr11:dup(q25), 0.63 Mb
10	21	17^+5^	Abnormal AFP MOM	Chr18:dup(0.1 Mb-13 Mb), 13 Mb	Refused	Chr18:dup(p11.32p11.21), 14.79 Mb
11	34	16^+0^	Only NIPT	Chr11:dup(56 Mb-133 Mb), 78 Mb	46,XN	Chr16:dup(p11.2), 1 Mb
12	39	17^+1^	Advanced maternal age	Chr8:dup(116 Mb-129 Mb), 14 Mb	Refused	Chr8:dup(q23.3q24.12), 4.24 Mb; Chr17:dup(q21.32q22), 4.65 Mb
13	29	19^+6^	Only NIPT	Chr18:dup(0.1 Mb-11 Mb), 11 Mb	46,XN	No abnormalities
14	30	16^+4^	Only NIPT	Chr15:del(38 Mb-100 Mb), 62 Mb	46,XN	No abnormalities
15	35	16^+5^	Only NIPT	Chr20:del(3 Mb-60 Mb), 57 Mb	46,XN	No abnormalities
16	33	17^+4^	Serological screening indicated low risk	Chr20:del(33 Mb-61 Mb), 28 Mb	46,XN	No abnormalities

*NIPT* noninvasive prenatal testing, *CNV* copy number variation, *MoM* multiple of the median, *AFP* alpha fetoprotein

### Sex chromosome abnormalities

The NIPT results of this study showed 17 cases of fetal sex chromosome abnormalities, 11 of which were confirmed by amniocentesis (Table 4). The coincidence rate was 64.7%. Among the 6 cases with inconsistent results, 5 were 45,X chromosomal abnormalities, as indicated by NIPT, and 1 was an 47,XYY abnormality. Among the 17 cases that showed sex chromosome abnormalities, 1 case of serological screening showed a high risk of trisomy 21; 3 cases of serological screening showed abnormal MoM; 1 case of serological screening showed a low risk; and 4 cases were pregnant women with advanced maternal age. Among the 48 fetuses with chromosomal abnormalities indicated by NIPT, 32 cases were confirmed by amniocentesis. The coincidence rate was 66.7%. The remaining NIPT false positive cases resulted in the continuation of the pregnancy, and no abnormalities were found during follow-up. All abnormal microdeletion/microduplications were de novo.

**Table 4:** Analysis of 17 cases of fetal sex chromosome abnormality indicated by NIPT

No.	Age (years)	Gestational weeks	NIPT indication	NIPT results	Karyotype results
1	32	17^+1^	Only NIPT	45,X	No abnormalities
2	34	14^+6^	Only NIPT	45,X	No abnormalities
3	22	23^+3^	Only NIPT	47,XYY	No abnormalities
4	19	17^+4^	Abnormal AFP MOM	45,X	45,X[78]/46,XX[22]
5	34	17^+1^	Serological screening indicated low risk	45,X	45,X
6	38	20^+4^	Abnormal AFP MOM	47,XXY	47,XXY
7	32	16^+1^	Abnormal PAPP-A MOM	45,X	45,X
8	18	17^+6^	Only NIPT	45,X	45,X
9	35	14^+3^	Only NIPT	47,XXY	47,XXY
10	36	17^+1^	Advanced maternal age	45,X	No abnormalities
11	35	15^+0^	Only NIPT	47,XXY	47,XXY
12	29	22^+1^	Only NIPT	45,X	No abnormalities
13	34	17^+3^	Only NIPT	47,XXY	47,XXY
14	41	12^+4^	Advanced maternal age	47,XXY	47,XXY
15	30	18^+4^	Only NIPT	45,X	No abnormalities
16	41	16^+3^	Advanced maternal age	47,XXY	47,XXY
17	19	19^+1^	High risk of trisomy 21	47,XXY	47,XXY

*NIPT* noninvasive prenatal testing, *MoM* multiple of the median, *AFP* alpha fetoprotein, *PAPP*-*A* pregnancy associated plasma protein-A

## Discussion

At present, neonatal birth defects remain a worldwide problem, with many countries having a high incidence rate of these defects. The extensive development of traditional serological prenatal screening, which is currently the most common screening method used, has made a significant contribution to reducing the birth of fetuses with chromosomal abnormalities, such as chromosomal aneuploidy. However, even though all high risk pregnant women who receive traditional serological screening undergo invasive amniocentesis fetal karyotype analysis, there was still a 5 to 50% misdiagnosis rate in serological screening [11]. Traditional serological prenatal screening cannot be used to screen for other fetal chromosomal abnormalities. In addition, there have been reports that chromosomal deletions, chromosomal duplications, and sex chromosome abnormalities can be the causes of neonatal birth defects, mostly consisting of new mutations [12]. Compared to traditional serological screening, NIPT has been increasingly used for its advantages, such as its noninvasiveness, high detection rate, low false positive rate, wide range of pregnancy, lower clinical information, and relatively easy method of quality control [13]. Not only does NIPT have a higher detection rate for trisomy 21, trisomy 18, and trisomy 13, it also has a better detection rate for other chromosomal abnormalities. Previous research [14, 15] has shown that NIPT can detect microdeletions and microduplications of fetal genomes above 300 Kb. In the present study, NIPT technology was successfully used to detect 15 cases of fetal chromosomal deletions ranging from 1.5 Mb to 69 Mb, 12 of which were finally confirmed by amniocentesis. The CNV detection results of 10 cases were almost consistent with their NIPT results. Our previous research also showed that NIPT can detect microdeletion of approximately 5 Mb. CNV detection was used to further pinpoint specific deletion areas, confirming the results yielded by NIPT [16]. This finding was consistent with previous reports in the literature [17]. In addition, the NIPT results of 16 cases indicated duplicate fragments of fetal chromosomes, 9 of which were confirmed by amniocentesis. The results of 3 CNV detections showed different multiple segments microduplication sizes from those shown by NIPT. One of these CNV detection results also indicated a microduplication of chromosome 17 approximately 4.65 Mb in length. It appears that the repetition of multiple fragments can cause a false increase in the repeated fragments of NIPT results. The NIPT results of 1 case showed a 78 Mb duplication of chromosome 11, while CNV detection showed a microduplication of approximately 1 Mb in chromosome 16. In another case, the karyotype results were inconsistent with the NIPT results. This showed that NIPT has certain limitations with regard to the detection of repeated small fragments. However, NIPT can provide a good indication of chromosomal abnormality. Analysis of the test indicators of the 21 cases of NIPT indicating chromosomal deletion and duplication, confirmed by amniocentesis, revealed that 11 cases were pregnant women with advanced maternal age, 1 case of serological screening indicated a low risk, 1 case of serological screening indicated a high risk of trisomy 21, 1 case of serological screening revealed abnormal AFP MoM, and 1 case revealed NT thickening. Preliminary analysis results show that NIPT has a certain application value in detecting chromosomal deletions and duplications under the indications of advanced age, NT thickening, MoM abnormalities in serological screening. However, in view of the small amount of positive specimens, it is necessary to further increase the sample size analysis.

At present, prenatal screening of fetal sex chromosome abnormalities has posed a relative blind spot [18]. These cases are often missed due to the low incidence rate in early childhood; healthcare follow-up appointments tend to occur during this period, thus missing the best period for intervention and therapy. Because abnormal sex chromosomes are often accompanied by reproductive system damage and endocrine disorders, the risk of diabetes, heart disease, and tumor disease is also greatly increased in such cases [2, 19, 20]. Therefore, screening for fetal sex chromosome abnormalities is crucial. In the present study, NIPT was used to detect 17 cases of sex chromosome abnormalities, 11 of which were confirmed through amniocentesis. Among the 6 cases yielding inconsistent results, 5 had 45,X chromosomal abnormalities, as indicated by NIPT, and 1 had an 47,XYY abnormality. In this study, NIPT demonstrated certain interference with the detection of 45,X chromosome abnormalities. However, NIPT has good clinical application value for the detection of other sex chromosome abnormalities. Analysis of the NIPT test indicators of 11 verified cases of sex chromosome abnormalities showed that 1 case of serological screening indicated a high risk of trisomy 21; 3 cases of serological screening showed abnormal MoM, and 1 case of serological screening indicated a low risk. It can be seen that serological screening for MoM abnormalities seems to be more sensitive to sex chromosome abnormalities. Similar to the 2 cases with low risk serological screening results, if there was no further NIPT test, these fetuses with chromosomal abnormalities will be missed.

There are also some cases of NIPT false positive or inconsistencies with amniotic fluid karyotype results in this study. The cause of differences or false positives may be related to the following factors. Firstly, because some cases had been confirmed that the deficiencies or duplications were relatively small, it iwas difficult to find the deleted and duplicated chromosomes below 10 Mb in conventional karyotyping. Therefore, the amniotic fluid karyotype analysis may miss the detection, resulting in differences between the NIPT or CNV results and the karyotype. Secondly, if the guanine and cytosine content of the X chromosome is shifted, the high homology of the X and Y chromosomes is not conducive to discrimination, so the Y chromosome has more similar fragments than other chromosomes, resulting in a reduction in sequencing noise ratio, which may cause inaccurate analysis and a high false positive rate. Finally, confined placental mosaicism (CPM) is considered to be one of the causes of false positive NIPT. Placental cells with CPM include both normal cells and cells with abnormal chromosomes. The cell-free fetal DNA fragments in NIPT are mainly obtained from placental cells. Cells with abnormal chromosomes in the placenta will cause false positive NIPT results [21]. Therefore, NIPT has a higher detection rate as a screening method. But for pregnant women whose NIPT test is abnormal, further prenatal diagnosis is necessary.

In summary, due to its rapid development, NIPT can be applied not only in prenatal screening for chromosomal aneuploidy, but also has a good application value in terms of detecting chromosomal deletions, chromosomal duplications, sex chromosome abnormalities, and so on. NIPT is especially useful in the detection of microdeletion and microduplication and can thus make up for the deficiencies of amniotic fluid karyotype analysis, which are easy to miss when artificial visual judgment is used. Although some NIPT results are inconsistent with amniocentesis results, NIPT has a good screening value for chromosomal abnormalities.

### Limitations

Several limitations of the study should be considered. Firstly, This study failed to confirm the diagnosis of amniocentesis in all pregnant women with NIPT results indicating chromosomal abnormalities. Further, the results were biased to some degree because the pregnant women who refused to undergo amniocentesis karyotyping were not included in the study. Secondly, NIPT has a limited ability to detect structural chromosomal abnormalities, which may be missed or misdiagnosed to some extent. Therefore, invasive prenatal diagnosis is still needed to detect such abnormalities. Finally, there may be some false negative results of NIPT in this study, such as for those pregnancies that have sonographic abnormalities and normal NIPT results, those pregnancies might have a genetic abnormality under the NIPT test resolution and even monogenetic. These false negative results may be ignored and lead to fetuses with birth defects, which may require further follow-up and other testing strategies.

## Conclusion

NIPT, as a method of screening for fetal chromosomal abnormalities, has a very good application value in terms of improving the detection rate of fetal chromosomal abnormalities such as chromosomal deletions, chromosomal duplications, and sex chromosome abnormalities, although further prenatal diagnosis may be needed.

## Data Availability

The datasets generated and analyzed during the current study are available from the corresponding author on reasonable request.

## Abbreviations

NIPT: Noninvasive prenatal testing
CNV: Copy number variation
NT: Nuchal translucency
MoM: Multiple of the median
